# Audience-Specific Health Communication: Mixed Methods Evaluation of the Maria Ciência AI-Assisted Knowledge Translation Tool

**DOI:** 10.2196/78843

**Published:** 2026-03-03

**Authors:** Mariana Araújo-Pereira, Klauss Villalva-Serra Jr, Gustavo Pires-Ramos, Beatriz Sousa-Peres, Joanã Nascimento Conceição-Oliveira, Sarah Dourado Maiche, Rebeca Rebouças da Cunha Silva, Bruno de Bezerril Andrade

**Affiliations:** 1 Laboratório de Pesquisa Clínica e Translacional, Instituto Gonçalo Moniz, R Waldemar Falcão 121, Salvador, 40296-710, Brazil, 1 7131762022; 2 Universidade Federal da Bahia, Salvador, Brazil; 3 Curso de Mestrado de Tecnologias em Saúde, Escola Bahiana de Medicina e Saúde Pública, Salvador, Brazil

**Keywords:** science communication, custom GPT, public health, scientific literacy, education

## Abstract

**Background:**

Scientific misinformation remains a major barrier to effective health communication. Bridging the gap between academic research and public understanding requires tools that simplify scientific language and adapt content to diverse audiences.

**Objective:**

This study presents Maria Ciência (LPCT-IGM), a specialized GPT-based assistant for science communication. The tool supports researchers in translating peer-reviewed scientific findings through simple prompts into accessible, ethically appropriate materials tailored for children, the general public, health professionals, and policymakers.

**Methods:**

The tool was configured using prompt engineering techniques and guided by curated reference materials on inclusive and nonstigmatizing scientific language. Materials derived from 47 public health papers resulted in 188 outputs, which were assessed by 121 evaluators using 4 criteria: clarity, level of detail, language suitability, and content quality. In addition, outputs generated by Maria Ciência were compared with those produced by a base large language model and with human-written science communication materials. Readability and linguistic accessibility were assessed using multiple established metrics.

**Results:**

Worldwide, mean scores were high: clarity (4.90), language suitability (4.78), content quality (4.72), and level of detail (4.56), on a 5-point scale. Materials for children and the general public consistently achieved the highest ratings across all criteria. A targeted comparison with the base large language model demonstrated superior performance of Maria Ciência in contextual stability. Readability analyses indicated that Maria Ciência’s outputs were significantly more accessible than human-written texts, while maintaining high legibility classifications.

**Conclusions:**

Maria Ciência demonstrates the potential of artificial intelligence–assisted tools to enhance knowledge translation and counter scientific misinformation by producing scalable, audience-specific content that balances accessibility and informational integrity.

## Introduction

Scientific misinformation is one of the most pressing challenges of our time, with direct consequences for public trust, the implementation of health policies, and the protection of population health [1]. The COVID-19 pandemic sharply illustrated how inaccurate or distorted information can compromise collective responses, with measurable impacts on morbidity and mortality [2]. The term “infodemic,” adopted by the World Health Organization, refers to the overwhelming volume of both accurate and misleading content that undermines access to trustworthy guidance. This phenomenon is amplified by digital platforms and social media, where misinformation spreads faster than corrective content. Efforts to manage infodemics have become a policy priority for global and national institutions, particularly in response to phenomena such as vaccine hesitancy, which has been linked to the resurgence of diseases such as measles.

Addressing misinformation requires more than reactive fact-checking; it demands the proactive translation of scientific knowledge into accessible, contextually relevant communication. Effective communication must also be timely, audience-centered, and grounded in the social and cultural contexts of the target populations. However, the scientific community itself often struggles to engage effectively with nonspecialist audiences. The persistence of a publication-centered academic culture, combined with time constraints and a lack of training, limits researchers’ ability to participate in outreach activities [3]. While efforts to integrate science communication into academic curricula are increasing, there remains a need for structural support and practical tools to facilitate engagement beyond scholarly environments.

Recent advances in artificial intelligence (AI) have expanded its application beyond clinical decision support into health communication and knowledge translation. Capability- and function-oriented reviews of AI in health care have highlighted a growing shift toward systems designed to support information delivery, user engagement, and audience adaptation, rather than exclusively diagnostic or predictive tasks. These perspectives emphasize that the value of AI in health increasingly depends on how effectively systems align outputs with user needs, contexts, and levels of expertise. In parallel, research on explainable AI has underscored transparency, interpretability, and usability as central requirements for trust, acceptance, and ethical deployment, particularly in health-related applications [4]. Although explainable AI has been extensively discussed in the context of clinical decision support systems, its core principles are equally relevant for health communication tools that aim to produce clear, audience-appropriate, and trustworthy content.

This paper presents Maria Ciência: an AI-assisted platform designed to translate scientific content into tailored, accessible formats for diverse audiences. Developed using a custom GPT model, the tool supports researchers in generating science communication materials adapted for children, adults with low literacy, health professionals, and decision-makers. Importantly, Maria Ciência is not intended to function as a clinical decision support system, nor to generate diagnostic or therapeutic recommendations. Instead, it operates as an audience-facing knowledge translation tool, focused on mediating peer-reviewed scientific evidence into formats appropriate for different levels of literacy, expertise, and social context.

The platform was created with the goal of enhancing the reach and impact of health-related scientific information, particularly in contexts where misinformation can influence individual behaviors and public health outcomes. The approach integrates AI with ethical oversight, thematic supervision, and practical communication strategies, aligning with emerging efforts to design responsible, user-centered AI systems for health communication. In contrast to generic chatbot applications, Maria Ciência is grounded in bioethical principles, equity-driven design, and a commitment to cultural and educational inclusivity. By enabling the same scientific input to be transformed into multiple outputs, the platform offers a scalable and adaptable response to the challenges of misinformation and scientific inaccessibility in public health and beyond.

## Methods

### Study Design

This study presents the development and evaluation of Maria Ciência, an AI-powered assistant designed to translate peer-reviewed research into audience-specific communication products. The tool was developed with the objective of promoting inclusive, accurate, and culturally sensitive dissemination of scientific knowledge, particularly in the field of public health. The methodological approach combined AI-supported content generation, audience-specific language adaptation, and empirical evaluation based on stakeholder feedback.

### Creation and Structure of the Tool

Maria Ciência was developed on the ChatGPT Plus (OpenAI) platform, a commercial version of ChatGPT (OpenAI) that allows for the creation of customized GPTs (also known as AI assistants) through detailed configuration of instructions, role definitions, and operational parameters. The primary function of Maria Ciência is to enable researchers to input scientific materials, such as peer-reviewed papers, and select the intended target audience. Based on this input, the assistant generates adapted communication materials suitable for various reader profiles, including children, the general population, health professionals, and policy decision-makers. These outputs are designed to be practical for use in educational, clinical, and public outreach contexts. ChatGPT is an AI-generated content developed by OpenAI that uses a transformer decoder-only architecture. The GPT model used in this approach was ChatGPT 4.5, released in February 2025. This version stands out as one of the most current versions alongside the ChatGPT o4-mini version, achieving in accuracy tests a 62.5% correctness rate, the highest value in software quality tests among the versions, and the lowest hallucination rate at 37.1%.

### Prompt Configuration and Operational Guidelines

The Maria Ciência assistant was configured through prompt engineering techniques combined with a carefully curated set of reference documents that established linguistic, ethical, and stylistic parameters. This configuration aimed to guide the assistant in generating respectful, inclusive, and audience-appropriate outputs. Training the AI assistant with carefully selected documentation greatly improves the accuracy and relevance of its responses [5,6]. In this study, we used reference materials on scientific dissemination, inclusive languages, and health dictionaries to fine-tune the model. Of note, the tool was initially tested in Brazilian Portuguese.

The assistant was assigned the explicit role of a Specialized Science Communicator, with advanced knowledge in public health, immunology, infectious and chronic diseases, and health communication. Its core objective was to translate complex scientific knowledge into formats that could be readily understood by diverse audiences. The interaction protocol included three primary steps: (1) understanding user needs: prompting users to specify the target audience (children, general public, health students, or health managers), (2) continuous engagement: encouraging deeper interaction through follow-up questions, and (3) final content generation: producing the final adapted text aligned with the audience’s profile.

Content adaptation guidelines were explicitly defined for each audience segment: (1) children: playful and narrative-driven writing inspired by Writing for Young Minds, incorporating contextual summaries and storytelling techniques; (2) general public: simplification of scientific concepts with actionable health information, drawing from accessible Brazilian sources such as Superinteressante and Profissão Biotec; (3) health students: simplified explanations while maintaining technical terminology, with emphasis on key health concepts; and (4) health professionals and managers: structured summaries including population characteristics, methodological overviews, and actionable public health recommendations (typically 5 suggested policy improvements).

Additionally, for social media content, the assistant adapted outputs for platforms such as Instagram (Meta) and LinkedIn, ensuring appropriate tone, hashtags, and visual alignment for each context.

### Training Materials

Among the sources used were the “Na ponta das línguas: pequeno glossário para apoiar o enfrentamento do estigma e da discriminação,” carried out with the support of the Brazilian Ministry of Health, which offers terminology to reduce stigma in health communication [7], as well as the “UNAIDS Terminology Guidelines” [8], which provide recommendations for respectful and accurate language related to HIV and global health. The “Global TB Dictionary” was used to ensure technical accuracy in tuberculosis-related content, and also the “UNICEF Terminology Dictionary” for the protection of children regarding matters of a sexual nature [9]. To support outputs for younger audiences, the tool’s language was informed by resources such as “Writing for Young Minds” and adapted scientific texts such as those published in Frontiers for Young Minds [10]. Additional materials, including guides to science communication [11,12] and examples of accessible writing from Brazilian science outreach initiatives such as “Profissão Biotec” [13], were used to calibrate tone, clarity, and structure across all outputs.

The training process emphasized the use of nonstigmatizing and inclusive language, minimization of unnecessary technical jargon, clear structuring of information according to literacy level, and contextual sensitivity to cultural and ethical dimensions of health communication. An additional focus was placed on ensuring equitable representation and fairness in responses across diverse population groups, particularly those historically marginalized in public health communication. The outputs generated by Maria Ciência included short narratives and analogies for children, simplified papers for the general population, technical summaries for health professionals, strategic briefs for health managers, and communication materials designed for social media. Additionally, Maria Ciência includes, in its presentation on the question bar, preconfigured prompts for users with guiding questions for using the chatbot, such as “translate this article for the general public,” “how to explain this concept to children,” or “create an accessible summary for children.” Finally, the assistant was programmed to provide appropriate attribution for all generated materials, including the original scientific source, first author, journal, and year of publication.

### Generation of Outputs for Evaluation

Following the configuration of the assistant, we selected 47 peer-reviewed papers (on public health, infectious diseases, and epidemiology) from our institution to serve as the training material. This approach provided the opportunity to invite the original authors of these papers to participate as evaluators in the assessment team. To minimize potential evaluation bias, the chatbot outputs for each paper were generated by external collaborators who were not part of the research group and who did not have a scientific background. Using only the preconfigured guiding questions in the chatbot interface, these collaborators generated 4 outputs per paper, including “for children,” “for health managers,” “for social media,” and “for the general public,” resulting in a total of 188 outputs. All outputs were generated using a dedicated user account created exclusively for this purpose, to minimize potential bias from prior sessions or unrelated model interactions.

### Public Evaluation of Chatbot Outputs

Following the generation of 188 outputs (4 per paper across 47 selected papers), a public evaluation process was conducted to assess the quality and appropriateness of the content produced by Maria Ciência. A standardized online evaluation form was developed for this purpose, structured to allow systematic feedback from diverse audiences. The evaluation process engaged 5 stakeholder groups: authors of the original papers, health professionals and students, social media specialists, members of the general public, and health managers. The evaluation form was disseminated through social media channels to reach a broad and heterogeneous audience. In addition, the original authors of the selected papers were invited to participate in the evaluation. This dual approach enabled the inclusion of both expert and lay perspectives in the assessment process. Participation was voluntary and anonymous.

Of note, evaluators were allowed to assess materials not exclusively targeted to their own audience profile to capture broader perceptions of clarity, tone, and appropriateness, reflecting real-world dissemination contexts in which health communication materials are often encountered by diverse audiences. In addition, children were not recruited as evaluators in this study, given that research involving minors requires parental permission and age-appropriate assent procedures. Given the exploratory nature of this evaluation and the absence of a child-centered protocol, the assessment of child-focused materials relied on adult evaluators.

Each participant was asked to assess selected chatbot outputs according to the following criteria: (1) Clarity: Is the text clear and appropriate for the intended audience? (2) Detail: Does the text provide sufficient and relevant information? (3) Language suitability: Is the language appropriate for the literacy level and context of the intended audience? (4) Content quality: Does the text maintain scientific accuracy and communicative effectiveness?

Participants assigned scores on a scale from 1 (poor) to 5 (excellent) for each criterion. Additionally, the form included an open field for qualitative comments, enabling participants to provide contextual feedback on strengths, limitations, or suggestions for improvement. Qualitative feedback provided by anonymous evaluators was analyzed using thematic categorization. Comments were grouped into four predefined domains: (1) language (clarity, accessibility, and appropriateness of language use); (2) information (accuracy, level of detail, and appropriateness of content for the audience); (3) structure (organization, narrative flow, and format of the material); and (4) proposal (whether the material complied with the intended purpose and target audience). For each domain, comments were further classified as either criticism or praise (for language, information, and structure), or as complies with proposal or does not comply with proposal (for proposal). Each comment was reviewed independently and could be assigned to multiple categories when it addressed more than one thematic domain.

### Accuracy Evaluation and Comparison With Base GPT

In addition to the public evaluation of the outputs, a focused accuracy evaluation was conducted to compare the performance of Maria Ciência with that of the base GPT-4.5 (OpenAI) model. This comparison aimed to assess whether the custom configuration of Maria Ciência, which incorporates training on inclusive, nonstigmatizing, and health-appropriate language, enhanced the model’s ability to maintain contextual relevance and communicative precision in public health content generation. For this purpose, a selected set of questions previously answered by Maria Ciência was resubmitted to both Maria Ciência and the base GPT-4.5 (without the custom configuration) separately.

To minimize potential bias from session memory, priming, or model adaptation effects, the evaluations for Maria Ciência and base GPT-4.5 were conducted using separate user accounts. This ensured that the comparative responses were generated independently, reducing the risk of inadvertent learning from previous interactions within the same account. In addition, to assess response stability, the same questions were submitted multiple times in different conversational sequences, allowing us to evaluate whether the models maintained contextual coherence across repeated interactions.

Responses from both models were evaluated by a team composed of health care professionals and undergraduate students. The evaluation criteria included 4 key dimensions: whether the response accurately established the context of the question; whether contextual coherence was preserved throughout the conversation; whether there was any interruption or drift from the intended context; and, if such drift occurred, whether the model was able to recover and return to the appropriate context. Each dimension was scored on a qualitative scale from 1 (poor) to 5 (excellent). The comparative analysis of results aimed to determine whether Maria Ciência’s configuration effectively enhanced contextual accuracy and stability, thus supporting its suitability for reliable and ethically appropriate use in public health communication. The following four dimensions were used to structure the assessment: (1) Establishment of a context: Does the answer fulfill the objective of the question according to the inserted context? (2) Continuity of conversation without specific context of the question: Do the following answers in the conversation with the chatbot lose the context in relation to the question? (3) Interruption of context: Do the answers interrupt or stop fulfilling the context of the question? (4) Return to context: Even after the interruption of the context or digression, is the chatbot able to return to the context of the question?

The tool operates in various languages, having been tested by the developers at Maria Ciência in Portuguese, English, Spanish, Italian, and French. Moreover, as it is a GPT assistant, the platform supports more than 50 languages [14]. Regardless of the language, the generated content follows the same principles of technical configuration, linguistic curation, and thematic supervision. This version preserves the commitment to accessibility, scientific accuracy, and ethical adequacy in knowledge translation, with a view toward application in international and multilingual contexts.

### Comparative and Readability Analyses

We conducted additional analysis, including human-written science communication materials and outputs generated by a nonspecialized base GPT-4.5. First, we performed an active search for human-authored news papers and popular science texts related to the peer-reviewed papers previously included in the main evaluation. This search yielded human-written communication materials corresponding to 5 of the originally analyzed scientific papers, all published in open-access media outlets.

In addition, to include recent and independent examples, we searched Google News (on December 1, 2025) using the query “new study in infectious diseases.” From the results, we selected the first five news reports that met the following criteria: (1) publication in non–university-affiliated media outlets, (2) reference to peer-reviewed scientific papers, and (3) availability of the original scientific papers under open-access conditions.

For these five additional papers, audience-oriented texts were generated using both Maria Ciência and the base GPT-4.5 model. The same simple prompt was applied to the base model across all cases (“transform this article into a text for the general population”), allowing a direct comparison between specialized and nonspecialized large language model outputs.

All materials (Maria Ciência outputs, base GPT-4.5 outputs, and human-written texts) were subjected to a standardized textual readability analysis. This analysis was applied to texts generated for all target audiences and encompassed both the original evaluation set and the additional comparative corpus. Readability metrics included: Flesch reading ease, Gulpease index, Flesch-Kincaid grade level, adapted Gunning fog index, automated readability index (ARI), Coleman-Liau index, letter-to-word ratio, syllable-to-word ratio, words-per-sentence ratio, and proportion of complex words. All readability analyses were conducted using the ALT (analysis of language and text) software, a tool specifically developed for Portuguese-language texts, as described by Moreno et al [15] (2023). This approach enabled a quantitative and language-appropriate comparison of textual complexity across human-written materials and AI-generated outputs, independently of subjective evaluator impressions.

### Data Analysis

Quantitative data from the evaluation forms were analyzed using descriptive statistics. For each evaluation criterion, means and SDs were calculated. Frequencies and percentages were computed for the classification of evaluator identities and for the distribution of outputs across target audiences. All analyses were conducted using the structured database generated from the stakeholder evaluations. For comparative purposes, we compared each readability metric according to the text generator (Maria Ciência, base GPT-4.5, or human-written).

### Ethical Considerations

This study was conducted as a public opinion survey evaluating AI-generated science communication materials. All data were collected through voluntary and anonymous participation, without the collection of identifiable or sensitive personal information, and without any form of intervention or risk to participants. In line with international ethical standards and in accordance with Brazilian Resolution CNS 510/2016, which exempts public opinion research involving nonidentified participants from requiring formal ethics committee approval, this study did not require submission to a research ethics committee.

## Results

### Overview

Before launching the public evaluation, we first analyzed the process of generating the 188 outputs used for assessment. The generation was conducted by external collaborators with no scientific background, using a dedicated user account created exclusively for this purpose. During this process, important differences were observed between the use of the free version and the paid version (ChatGPT Plus). In the free version, the model frequently (10%) exhibited technical limitations: it would often require questions to be reformulated and occasionally produce incomplete outputs. The average generation time per output in this version was approximately 15.2 (SD 2.3) seconds. After upgrading to the ChatGPT Plus version, performance improved in measurable operational terms. The model produced responses more rapidly and with greater consistency, showing a more direct communication style. In this version, the mean generation time decreased to 8.5 (SD 1.5) seconds, and the incidence of incomplete outputs was eliminated. Despite these differences, the free version remained capable of generating the requested outputs using the predefined prompts provided by Maria Ciência; however, it required longer processing time and occasional manual resubmission of prompts to ensure complete responses.

### Evaluator Profile and Distribution of Reviewed Outputs

**Figure 1.**
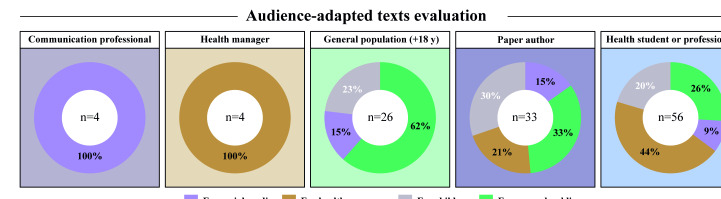
Donut charts summarizing the types of audience-adapted texts proportionally reviewed by each participant identity classification. Segment colors represent the percentage of texts tailored for different audiences, including (1) for social media (lavender), (2) for health managers (golden), (3) for children (gray), and (4) for the general public (green). Each donut reflects the distribution of text types reviewed by participants within a specific identity group.

The evaluation of Maria Ciência involved 121 responses to the form, stratified across 5 distinct groups, each representing a key target audience of the tool. The distribution of respondents was as follows: health professionals and students (56/121, 44.6%), original authors of the scientific papers (33/121, 27.3%), members of the general population (26/121, 21.5%), communication specialists (4/121, 3.3%), and health managers (4/121, 3.3%; Figure 1). Each evaluator assessed outputs generated for one or more specific target audiences, including “for children” (n=27), “for health managers” (n=35), “for social media” (n=24), and “for the general public” (n=35). Among these groups, only the original paper authors had prior in-depth knowledge of the scientific content being communicated. Notably, Figure 1 illustrates the distribution of reviewed text types according to identity profile classification, revealing clear patterns in how different stakeholder groups engaged with the audience‐adapted outputs. Communication professionals and health managers exclusively reviewed solely the texts targeted at their respective profiles (100%), while the other evaluator profiles diversified their reviewed texts. Given the small proportion of the evaluator sample, results related to these subgroups were reported descriptively and were interpreted as exploratory.

Among the general population evaluators, 62% (16/26) assessed the “for the general public” texts, while 23% (6/26) provided feedback on “for children” versions, and 15% (4/26) on “for health managers.” Notably, none of the general population participants reviewed the “for social media” outputs. Health professionals and students provided a broader distribution of feedback, with 44% (24/56) evaluating “for health managers” outputs, 25% (14/56) “for the general public,” 20% (11/56) “for children,” and 9% (5/56) “for social media.” Finally, the paper authors demonstrated a balanced engagement across all 4 categories: 33% (11/33) reviewed “for the general public” outputs, 30% (10/33) “for children,” 21% (7/33) “for health managers,” and 15% (5/33) “for social media” (Figure 1). The distribution of respondents and their evaluation profiles across different target audiences is summarized in Table 1.

**Table 1.** Distribution of reviewer identities and rating frequencies by target audience. This table displays the frequency (n) and percentage (%) of reviewers’ self‐reported identity classifications and rating scores across 4 different domains, stratified by the number of reviews attributed to each target audience texts. *P* values were calculated using Fisher exact test (for nominal categorical counts) or Kruskal-Wallis tests (for ordinal rating distributions), comparing across the 4 target‐audience groups. A *P* value <.05 denotes a statistically significant difference among audiences.

	For children (n=27)	For health managers (n=35)	For social media (n=24)	For the general public (n=35)	*P* value
Variable					
Participant classification, n (%)					<.001
Paper author	10 (37)	7 (20)	11 (45.8)	5 (14.3)	
Communication professional	0 (0)	0 (0)	4 (16.7)	0 (0)	
General population (18+ years)	6 (22.2)	0 (0)	4 (16.7)	16 (45.7)	
Health manager	0 (0)	4 (11.4)	0 (0)	0 (0)	
Health student or professional	11 (40.7)	24 (68.6)	5 (20.8)	14 (40)	
Rating					
Clarity, n (%)					.21
1	0 (0)	0 (0)	0 (0)	0 (0)	
2	0 (0)	1 (2.9)	0 (0)	0 (0)	
3	0 (0)	2 (5.7)	0 (0)	0 (0)	
4	4 (14.8)	6 (17.1)	4 (16.7)	1 (2.9)	
5	23 (85.2)	26 (74.3)	20 (83.3)	34 (97.1)	
Detailing, n (**%**)					.28
1	0 (0)	0 (0)	0 (0)	0 (0)	
2	0 (0)	1 (2.9)	0 (0)	1 (2.9)	
3	2 (7.4)	8 (22.9)	4 (16.7)	1 (2.9)	
4	6 (22.2)	7 (20)	6 (25)	5 (14.3)	
5	19 (70.4)	19 (54.3)	14 (58.3)	28 (80)	
Language adequacy, n (%)					.20
1	0 (0)	0 (0)	0 (0)	0 (0)	
2	0 (0)	1 (2.9)	0 (0)	0 (0)	
3	3 (11.1)	1 (2.9)	2 (8.3)	1 (2.9)	
4	4 (14.8)	12 (34.3)	2 (8.3)	6 (17.1)	
5	20 (74.1)	21 (60)	20 (83.3)	28 (80)	
Content quality, n (%)					.11
1	0 (0)	0 (0)	0 (0)	0 (0)	
2	0 (0)	0 (0)	0 (0)	0 (0)	
3	0 (0)	4 (11.4)	1 (4.2)	0 (0)	
4	7 (25.9)	12 (34.3)	7 (29.2)	7 (20)	
5	20 (74.1)	19 (54.3)	16 (66.7)	28 (80)	

### Public Evaluation of Audience-Adapted Outputs

Each output was independently evaluated by respondents from multiple stakeholder groups, using 4 evaluation criteria: clarity of the text, level of detail, suitability of language for the intended audience, and overall content quality. Participants rated each criterion on a 5-point scale, with 5 indicating strong agreement regarding the quality or appropriateness of the item assessed. The distribution of evaluator profiles and scoring frequencies is presented in Table 1.

Within this exploratory evaluation, the adapted texts were rated highly across all criteria (Table 1). Texts targeting the general public received strong evaluations. Among members of this audience, mean scores were 4.94 (SD 0.25) for clarity, 4.56 (SD 0.89) for detail, 4.62 (SD 0.62) for language suitability, and 4.75 (SD 0.45) for overall quality. Students and health professionals were equally enthusiastic, with scores of 5.00 (SD 0.00) for clarity, 4.86 (SD 0.36) for detailing, 4.93 (SD 0.27) for language, and 4.93 (SD 0.27) for quality. Of note, researchers who authored the original papers were somewhat more critical, assigning 5.00 (SD 0.00) for clarity, 4.80 (SD 0.45) for detail, 4.80 (SD 0.45) for language, and 4.60 (SD 0.55) for overall quality (Table 2).

Similarly, the child-focused outputs were well received. The general public assigned near-perfect ratings of 5.00 (SD 0.00) for clarity, 4.83 (SD 0.41) for detail, 4.83 (SD 0.41) for language suitability, and 4.83 (SD 0.41) for content quality. Students and health professionals also rated the child-focused content highly, with a means of 4.82 (SD 0.40) for clarity, 4.64 (SD 0.67) for detailing, 4.73 (SD 0.65) on language suitability, and 4.73 (SD 0.47) regarding overall quality. Again, the original authors were more reserved, assigning 4.80 (SD 0.42) for clarity, 4.50 (SD 0.71) for detail, 4.40 (SD 0.84) for language, and 4.70 (SD 0.48) for overall quality (Table 2).

Next, the version tailored for health managers was rated lower by managers themselves, who rated it 4.00 (SD 1.41) for clarity, 4.00 (SD 1.41) for detail, 4.00 (SD 0.82) for language suitability, and 4.25 (SD 0.96) for content quality. Students and health professionals provided comparably high scores, with 4.83 (SD 0.48) for clarity, 4.58 (SD 0.72) for detail, 4.62 (SD 0.71) for language suitability, and 4.67 (SD 0.56) for overall quality. Original paper authors again expressed greater variability in their evaluations, especially regarding level of detail, with a mean of 3.29 (SD 0.49), similarly lower ratings were reported for clarity, which was 4.29 (SD 0.76), 4.43 (SD 0.53) for language, and 3.71 (SD 0.49) for content quality (Table 2).

For evaluations aimed at social media texts, all evaluators rated these texts highly. General public reviewers rated this version at 5.00 (SD 0.00) for clarity, 4.75 (SD 0.50) for detail, 4.50 (SD 1.00) for language suitability, and 4.50 (SD 1.00) for overall quality. Students and health professionals similarly gave high scores, 5.00 (SD 0.00) for clarity, 4.40 (SD 0.89) for detail, 4.80 (SD 0.45) for language suitability, and 4.60 (SD 0.55) for content quality. Communication professionals assigned a perfect mean score of 5.00 (SD 0.00) across all 4 criteria. As observed for other target audiences, paper authors provided more conservative ratings, with scores of 4.64 (SD 0.50) for clarity, 4.09 (SD 0.83) for detail, 4.73 (SD 0.65) for language suitability, and 4.55 (SD 0.52) for overall quality (Table 2).

**Figure 2.**
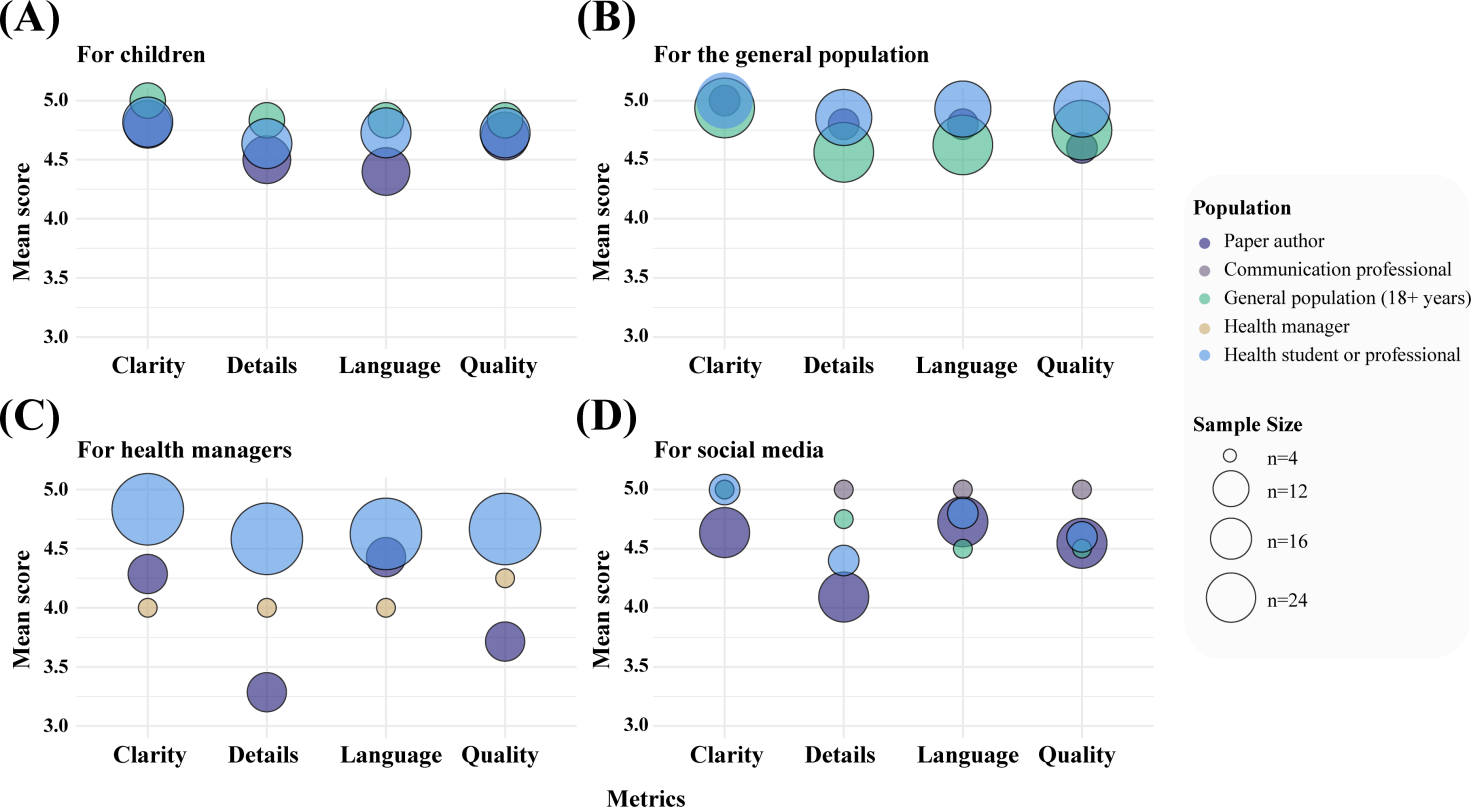
Mean evaluation scores of audience-adapted texts by criterion and evaluator population. Bubble plot presenting the mean scores for each evaluation criterion, clarity, detailing, language adequacy, and content quality, across four types of audience-adapted texts: (A) for children, (B) for the general population, (C) for health managers, and (D) for social media. Each colored bubble represents a different population of evaluators, as indicated in the legend on the right. The size of each bubble is proportional to the number of respondents from that population who rated the corresponding question for each text type.

**Figure 3.**
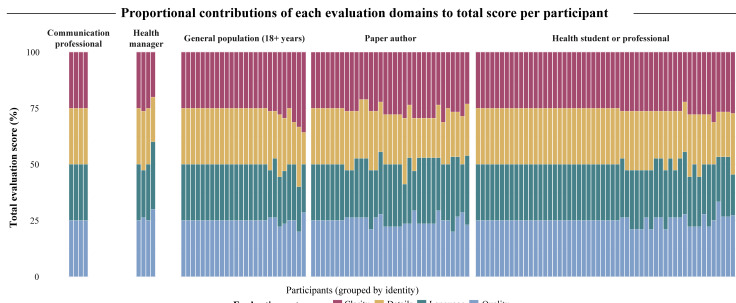
Proportional contributions of evaluation domains to each participant’s total score, grouped by reviewer identity. A 100% stacked bar chart in which each vertical bar represents an individual participant’s normalized total evaluation score (scaled to 100%), with participants organized along the x-axis and grouped by self-reported identity: paper author, communication professional, general population (18+ years), health manager, and health student or professional. Within each bar, colored segments depict the relative weight of each of the 4 evaluation categories, quality (light blue), language (teal), detailing (gold), and clarity (maroon), in that participant’s overall total rating (sum of individual 1-5 domain scores). The y-axis indicates the percentage contribution of each category to the participant’s total score.

Taken together, within this exploratory evaluation, audience-adapted texts received high mean scores across all 4 assessed criteria, regardless of the target audience. Notably, texts adapted for children and for the general population received particularly high ratings across all dimensions, with most mean scores approaching the maximum value of 5. Health students or professionals, communication professionals, and the general adult population tended to assign higher scores overall, while health managers and paper authors demonstrated greater variability in their assessments, especially for detailing of texts targeting health managers or social media platforms (Figure 2). Among all groups, paper authors exhibited the greatest variability in their evaluations. These patterns indicate broad perceived acceptability of the materials in this sample, with subtle differences in perceived quality depending on the evaluator profile (Figure 3).

**Table 2.** Average (SD) evaluation scores by audience and stakeholder group for each adapted output. This table presents the central tendency and dispersion (mean, SD) of four evaluation domains: (1) clarity, (2) detailing, (3) language suitability, and (4) content quality, across four target‐audience contexts (“for children,” “for health managers,” “for social media,” and “for the general public”). Within each context, scores are shown separately for each evaluator subgroup (authors, general public, students or health professionals, health managers, and communication professionals). All ratings were provided on a 5‐point scale ranging from 1‐5.

Target audience and evaluator	Clarity	Detailing	Language suitability	Content quality
For the general public, mean (SD)				
Authors	5.00 (0.00)	4.80 (0.45)	4.80 (0.45)	4.60 (0.55)
General public	4.94 (0.25)	4.56 (0.89)	4.62 (0.62)	4.75 (0.45)
Students or health professionals	5.00 (0.00)	4.86 (0.36)	4.93 (0.27)	4.93 (0.27)
For children, mean (SD)				
Authors	4.80 (0.42)	4.50 (0.71)	4.40 (0.84)	4.70 (0.48)
General public	5.00 (0.00)	4.83 (0.41)	4.83 (0.41)	4.83 (0.41)
Students or health professionals	4.82 (0.40)	4.64 (0.67)	4.73 (0.65)	4.73 (0.47)
For health managers, mean (SD)				
Authors	4.29 (0.76)	3.29 (0.49)	4.43 (0.53)	3.71 (0.49)
Students or health professionals	4.83 (0.48)	4.58 (0.72)	4.62 (0.71)	4.67 (0.56)
Health managers	4.00 (1.41)	4.00 (1.41)	4.00 (0.82)	4.25 (0.96)
For social media, mean (SD)				
Authors	4.64 (0.50)	4.09 (0.83)	4.73 (0.65)	4.55 (0.52)
General public	5.00 (0.00)	4.75 (0.50)	4.50 (1.00)	4.50 (1.00)
Students or health professionals	5.00 (0.00)	4.40 (0.89)	4.80 (0.45)	4.60 (0.55)
Communication professionals	5.00 (0.00)	5.00 (0.00)	5.00 (0.00)	5.00 (0.00)

In addition to quantitative ratings, anonymous evaluators were invited to provide open-ended comments on the chatbot-generated materials. A total of 68 comments were collected, distributed across the evaluated target audiences: “for children” (n=14), “for the general public” (n=27), “for health managers” (n=11), and “for social media” (n=16; Table 3). Across all categories, the overall tone of the comments was positive and constructive. For children’s materials, praise regarding language was the most frequent (8/14, 57.1%), while 42.9% (6/14) of comments did not address language. Information praise (4/14, 28.6%) and information criticism (4/14, 28.6%) were also observed. Only 7.1% (1/14) of comments suggested the material did not fully comply with its intended proposal. For health manager materials, comments were more evenly distributed: information criticism (4/11, 36.3%), language praise (4/11, 36.3%), and proposal compliance (9/11, 81.8%) were most common, though 18.2% (2/11) indicated noncompliance with the proposal. For social media materials, the highest frequency of feedback related to information criticism (6/16, 37.5%) and structure criticism (4/16, 25%), reflecting the platform-specific communication challenges. A majority (13/16, 81.3%) of comments judged the materials as compliant with the intended proposal. For general public materials, language praise was dominant (18/27, 66.7%), with a smaller proportion of comments addressing information (only 29.6% provided any information-related feedback). Most comments (23/27, 85.2%) indicated that the outputs complied with the intended proposal. Across all targets, the high proportion of proposal compliance comments indicates strong overall alignment between the outputs and their target audiences. A full compilation of the anonymous comments is provided in Multimedia Appendix 1.

**Table 3.** Distribution of types of comments by target audience. This table displays the frequency (n) and percentage (%). Comments were categorized into 4 domains: language, information, structure, and proposal. For each domain, comments were classified as criticism, praise, or not applicable (for language, information, and structure), or as complies with proposal or does not comply with proposal (for proposal). Comments could be assigned to multiple domains and categories.

Category	For children (n=14)	For health managers (n=11)	For social media (n=16)	For the general public (n=27)
Language, n (%)				
Criticism	0 (0)	1 (9.1)	2 (12.5)	2 (7.4)
Praise	8 (57.1)	4 (36.3)	5 (31.2)	18 (66.7)
Not applicable	6 (42.9)	6 (54.6)	9 (56.3)	7 (25.9)
Information, n (%)				
Criticism	4 (28.6)	4 (36.3)	6 (37.5)	5 (18.5)
Praise	4 (28.6)	2 (18.2)	1 (6.2)	3 (11.1)
Not applicable	6 (42.9)	5 (45.5)	9 (56.3)	19 (70.4)
Structure, n (%)				
Criticism	3 (21.4)	2 (18.2)	4 (25)	2 (7.4)
Praise	3 (21.4)	0 (0)	0 (0)	0 (0)
Not applicable	8 (57.1)	9 (81.8)	11 (68.8)	25 (92.6)
Proposal, n (%)				
Does not comply with the proposal	1 (7.1)	2 (18.2)	3 (18.7)	4 (14.8)
Complies with the proposal	13 (92.9)	9 (81.8)	13 (81.3)	23 (85.2)

### Comparative Accuracy and Context Stability Evaluation

A detailed comparison between Maria Ciência and the base GPT-4.5 model was conducted across 4 conversational criteria: establishment of context, continuity of conversation, resilience to interruption, and return to context, stratified by target audience (Table S1 in Multimedia Appendix 1). For Maria Ciência, mean scores were high across all target audiences and criteria. For the general public, Maria Ciência achieved perfect continuity of conversation (5.00, SD 0.00) and high stability across all other dimensions (establishment of context: 4.75, SD 0.50; interruption of context: 4.75, SD 0.50; and return to context: 4.75, SD 0.50). In comparison, the base GPT-4.5 model exhibited lower mean scores across several criteria.

For children, Maria Ciência again showed higher mean scores, with perfect scores for interruption of context (5.00, SD 0.00) and strong scores across other criteria (establishment of context: 4.66, SD 0.58; continuity: 4.66, SD 0.58; and return to context: 4.66, SD 0.58). The base GPT-4.5 model showed substantially lower performance in this category, particularly in continuity (3.00, SD 0.82) and establishment of context (3.25, SD 0.50), indicating challenges in maintaining audience-appropriate conversation flow for younger users.

For social media outputs, scores remained high, with means of 4.50 (SD 0.58) for establishment of context and continuity of conversation, and 5.00 (SD 0.00) for criterion resilience to interruption. In contrast, the base GPT-4.5 model exhibited greater variability, particularly in outputs for children and health managers. For children, mean scores were notably lower in the establishment of context (3.25, SD 0.96), continuity of conversation (3.00, SD 0.82), and resilience to interruption (3.00, SD 0.82), highlighting difficulties in maintaining and recovering conversational context. For health managers, while context establishment remained high (4.75, SD 0.50), performance dropped in criteria resilience to interruption (3.75, SD 0.96) and continuity of conversation (4.25, SD 0.50). Across all targets, the base GPT-4.5 model showed more frequent context drift and reduced continuity compared to Maria Ciência (Table S1 in Multimedia Appendix 1).

### Readability Evaluation

Readability metrics derived from the ALT framework revealed significant differences between texts generated by Maria Ciência and those generated by the base GPT-4.5 model. For materials designed for children, the overall grade level was higher for Maria Ciência outputs compared to base GPT-4.5 (MC: 8.23, SD 0.96 vs GPT: 7.56, SD 1.03; *P*=.002). While all texts from both generators were classified as having high legibility, differences emerged across several quantitative readability indices. Maria Ciência texts showed lower Flesch reading ease scores (MC: 67.1, SD 5.51 vs GPT: 71.3, SD 5.80; *P*=.001) and higher Flesch-Kincaid grade levels (MC: 7.58, SD 0.98 vs GPT: 6.70, SD 0.98; *P*<.001), indicating increased textual complexity. Similar patterns were observed for the adjusted Gunning fog index (*P*<.001), the ARI (*P*=.004), the number of words per sentence (*P*<.001), and several syllables per word (*P*=.002). No statistically significant differences were observed between generators for the Coleman-Liau index, letters per word, or proportion of complex words (Table 4).

**Table 4.** Readability analysis of texts. This table presents the central tendency and dispersion (mean, SD) of readability metrics, as well as the frequency (n) and percentage (%) for categorical variables.

	GPT base	Maria Ciência	*P* value
For children			
Overall grade level, mean (SD)	7.56 (1.03)	8.23 (0.96)	.002
Recommended age group (year), n (%)			.08
11‐14	39 (83)	30 (63.8)	
15‐18	8 (17)	16 (36.2)	
Legibility result, n (%)			—^a^
High	47 (100)	47 (100)	
Flesch reading ease, mean (SD)	71.3 (5.80)	67.1 (5.51)	.001
Gulpease index, mean (SD)	69.6 (4.26)	1104 (6883)	.32
Flesch-Kincaid grade level, mean (SD)	6.70 (0.98)	7.58 (0.98)	<.001
Adjusted Gunning fog index, mean (SD)	8.12 (0.85)	8.85 (0.79)	<.001
Automated readability index, mean (SD)	6.34 (1.24)	7.09 (1.18)	.004
Coleman-Liau index, mean (SD)	9.23 (1.21)	9.64 (1.05)	.09
Letters per word, mean (SD)	4.65 (0.21)	4.68 (0.20)	.51
Syllables per word, mean (SD)	1.98 (0.08)	2.03 (0.07)	.002
Words per sentence, mean (SD)	11.2 (1.44)	12.5 (1.45)	<.001
Complex words (%), mean (SD)	13.7 (3.44)	14.6 (2.44)	.15
For the health manager			
Overall grade level, mean (SD)	13.7 (1.70)	14.4 (2.05)	.11
Recommended age group (year), n (%)			.13
15‐18	10 (21.3)	5 (10.6)	
Can be easily understood by university students	30 (63.8)	35 (74.5)	
For people with a college degree	7 (14.9)	3 (6.4)	
Extremely difficult text	0 (0)	4 (8.5)	
Legibility result, n (%)			.32
High	10 (21.3)	5 (10.6)	
Medium	34 (72.3)	37 (78.7)	
Low	3 (6.4)	5 (10.6)	
Flesch reading ease, mean (SD)	27.8 (8.13)	32.6 (8.61)	.008
Gulpease index, mean (SD)	53.7 (6.72)	50.0 (4.36)	.003
Flesch-Kincaid grade level, mean (SD)	13.5 (1.81)	14.0 (2.22)	.24
Adjusted Gunning fog index, mean (SD)	11.5 (2.20)	14.0 (2.81)	<.001
Automated readability index, mean (SD)	13.3 (2.06)	14.2 (2.64)	.08
Coleman-Liau index, mean (SD)	16.4 (1.21)	15.0 (1.11)	<.001
Letters per word, mean (SD)	5.95 (0.21)	5.59 (0.22)	<.001
Syllables per word, mean (SD)	2.56 (0.10)	2.42 (0.11)	<.001
Words per sentence, mean (SD)	13.5 (4.17)	19.2 (6.09)	<.001
Complex words (%), mean (SD)	25.9 (3.44)	23.9 (3.08)	.006
For social media			
Overall grade level, mean (SD)	8.89 (2.05)	11.2 (1.59)	<.001
Recommended age group (years), n (%)			<.001
11‐14	20 (42.6)	1 (2.1)	
15‐18	24 (51)	40 (85.1)	
Can be easily understood by university students	3 (6.4)	6 (12.8)	
Legibility result, n (%)			.22
High	43 (91.5)	39 (83)	
Medium	4 (8.5)	8 (17)	
Flesch reading ease, mean (SD)	65.5 (12.8)	50.7 (8.95)	<.001
Gulpease index, mean (SD)	64.4 (6.92)	57.1 (4.27)	<.001
Flesch-Kincaid grade level, mean (SD)	8.02 (2.26)	10.7 (1.75)	<.001
Adjusted Gunning fog index, mean (SD)	9.49 (1.72)	10.9 (1.95)	<.001
Automated readability index, mean (SD)	7.91 (2.52)	10.7 (1.97)	<.001
Coleman-Liau index, mean (SD)	10.1 (2.20)	12.4 (1.50)	<.001
Letters per word, mean (SD)	4.76 (0.38)	5.14 (0.29)	<.001
Syllables per word, mean (SD)	2.03 (0.16)	2.20 (0.12)	<.001
Words per sentence, mean (SD)	13.5 (2.97)	16.0 (4.10)	.002
Complex words (%), mean (SD)	15 (4.83)	16.2 (3.12)	.17
For the general public			
Overall grade level, mean (SD)	10.5 (1.22)	9.96 (1.00)	.026
Recommended age group (year), n (%)			.52
11‐14	3 (6.4)	2 (4.3)	
15‐18	41 (87.2)	44 (93.6)	
Can be easily understood by university students	3 (6.4)	1 (2.1)	
Legibility result, n (%)			.31
High	44 (93.6)	46 (97.9)	
Medium	3 (6.4)	1 (2.1)	
Flesch reading ease, mean (SD)	53.1 (7.35)	58.7 (5.87)	<.001
Gulpease index, mean (SD)	59.5 (4.05)	60.5 (3.08)	.21
Flesch-Kincaid grade level, mean (SD)	9.92 (1.24)	9.33 (1.05)	.02
Adjusted Gunning fog index, mean (SD)	9.96 (1.23)	10.4 (1.11)	.07
Automated readability index, mean (SD)	9.83 (1.47)	9.02 (1.21)	.06
Coleman-Liau index, mean (SD)	12.2 (1.39)	10.9 (1.00)	<.001
Letters per word, mean (SD)	5.14 (0.25)	4.86 (0.18)	<.001
Syllables per word, mean (SD)	2.20 (0.11)	2.10 (0.08)	<.001
Words per sentence, mean (SD)	14.0 (2.38)	15.1 (1.83)	.02
Complex words (%), mean (SD)	16.2 (4.51)	15.8 (3.06)	.62

a Not available.

The readability analyses of texts generated for health managers revealed overall comparable patterns between Maria Ciência and the base GPT-4.5 model, with few statistically significant differences across metrics. The overall grade level did not differ significantly between generators (*P*=.11), as well as the classification by recommended audience level (*P*=.13), and the overall legibility categories (*P*=.32). Despite these similarities, Maria Ciência texts exhibited higher Flesch reading ease scores compared to base GPT-4.5 (MC: 32.6, SD 8.61 vs GPT: 27.8, SD 8.13; *P*=.008), higher Gunning fog index (MC: 14.0, SD 2.81 vs GPT: 11.5, SD 2.20; *P*<.001), alongside lower Gulpease index values (MC: 50.0, SD 4.36 vs GPT: 53.7, SD 6.72; *P*=.003), if compared to base GPT 4.5 model (Table 4). At the lexical and syntactic levels, Maria Ciência texts contained fewer letters per word (*P*<.001) and fewer syllables per word (*P*<.001), but substantially longer sentences (*P*<.001). The proportion of complex words was slightly lower in Maria Ciência outputs (23.9%, SD 3.08% vs 25.9%, SD 3.44%; *P*=.006; Table 4).

For materials intended for social media, Maria Ciência outputs exhibited a substantially higher overall grade level compared to base GPT-4.5 (MC: 11.2, SD 1.59 vs GPT: 8.89, SD 2.05; *P*<.001). Consistent with this pattern, having high legibility across multiple recommended age group classifications differed significantly between generators (*P*<.001). While base GPT-4.5 outputs were more frequently classified as suitable for younger adolescents (11‐14 y), the majority of Maria Ciência’s texts were classified as appropriate for older adolescents (15‐18 y). Despite these differences in textual complexity, overall legibility categories (high vs medium) were comparable between generators (*P*=.22), with most texts in both groups classified as having high legibility. Across multiple readability indices, Maria Ciência’s texts consistently demonstrated increased linguistic complexity. These outputs showed lower Flesch reading ease scores (MC: 50.7, SD 8.95 vs GPT: 65.5, SD 12.8; *P*<.001) and lower Gulpease index values (MC: 57.1, SD 4.27 vs GPT: 64.4, SD 6.92; *P*<.001), alongside higher Flesch-Kincaid grade levels (MC: 10.7 (SD 1.75 vs GPT: 8.02, SD 2.26; *P*<.001). Similar trends were observed for the adjusted Gunning fog index, ARI, and Coleman-Liau index (*P*<.001). At the lexical and syntactic levels, Maria Ciência outputs contained longer words, reflected by higher letters-per-word and syllables-per-word ratios (both *P*<.001), as well as longer sentences (*P*=.002). The proportion of complex words did not differ significantly between generators (Table 4).

Readability analyses indicated modest but statistically significant differences between texts generated by Maria Ciência and those generated by the base GPT-4.5 model for materials aimed at the general population. Maria Ciência outputs exhibited a slightly lower overall grade level compared to base GPT-4.5 (MC: 9.96, SD 1.00 vs GPT: 10.5, SD 1.22; *P*=.03). Recommended age group classifications were similar between generators (*P*=.52), with the majority of texts in both groups classified as suitable for adolescents aged 15‐18 years. Overall legibility categories (high vs medium) were also comparable (*P*=.31), with most texts classified as having high legibility. Several readability indices suggested improved linguistic accessibility in Maria Ciência outputs. These texts showed higher Flesch Reading Ease scores (MC: 58.7, SD 5.87 vs GPT: 53.1, SD 7.35; *P*<.001) and lower Flesch–Kincaid Grade Levels (MC: 9.33, SD 1.05 vs GPT: 9.92, SD 1.24; *P*=.02). The Coleman-Liau Index was also lower for Maria Ciência texts (MC: 10.9, SD 1.00 vs GPT: 12.2, SD 1.39; *P*<.001), indicating reduced lexical complexity. At the word level, Maria Ciência outputs contained fewer letters per word (MC: 4.86, SD 0.18 vs GPT: 5.14, SD 0.25; *P*<.001) and fewer syllables per word (MC: 2.10, SD 0.08 vs GPT: 2.20, SD 0.11; *P*<.001). In contrast, Maria Ciência texts exhibited a slightly higher number of words per sentence compared to base GPT-4.5 outputs (MC: 15.1, SD 1.83 vs 14.0, SD GPT: 2.38; *P*=.02). No statistically significant differences were observed between generators in the proportion of complex words (Table 4).

When directly compared with human-written texts, both base GPT-4.5 and Maria Ciência showed systematic and statistically significant differences across nearly all readability metrics. Human texts consistently exhibited higher overall grade levels (15.6, SD 1.96) than base GPT-4.5 (11.0, SD 1.15) and Maria Ciência (9.7, SD 0.67), indicating substantially greater linguistic complexity. This pattern was reinforced by multiple indices: human abstracts had lower Flesch reading ease scores and higher Flesch-Kincaid, Gunning fog, ARI, and Coleman-Liau indices, alongside longer sentences and a higher proportion of complex words (all with *P*<.01, Table 5). In contrast, both Maria Ciência and base GPT-4.5 generated texts that were easier to read, shorter at the sentence level, and lexically simpler. Importantly, while both systems differed from human writing in the same direction, Maria Ciência outputs tended to be consistently simpler than base GPT-4.5 across most metrics, including reading ease, grade level, sentence length, and lexical density (Table 5).

**Table 5.** Differences between Maria Ciência, base GPT 4.5, and human-written texts. This table presents the central tendency and dispersion (mean, SD) of readability metrics, as well as the frequency (n) and percentage (%) for categorical variables.

	GPT base°	Maria Ciência	Human	*P* value
Overall grade level, mean (SD)	11.0 (1.15)	9.70 (0.67)	15.6 (1.96)	<.001
Recommended age group, n (%)				<.001
11‐14 year	0 (0)	1 (10)	0 (0)	
15‐18 year	9 (90)	9 (90)	1 (10)	
Can be easily understood by university students	1 (10)	0 (0)	3 (30)	
For people with a college degree	0 (0)	0 (0)	6 (60)	
Legibility result, n (%)				<.001
High	9 (90)	10 (100)	1 (10)	
Medium	1 (10)	0 (0)	5 (50)	
Low	0 (0)	0 (0)	4 (40)	
Flesch reading ease, mean (SD)	50.6 (6.93)	58.7 (5.81)	32.4 (10.5)	<.001
Gulpease index, mean (SD)	57.8 (3.52)	60.9 (2.84)	47.1 (3.53)	<.001
Flesch-Kincaid grade level, mean (SD)	10.6 (1.10)	9.10 (0.77)	15.4 (1.92)	<.001
Adjusted Gunning fog index, mean (SD)	11.1 (1.43)	10.1 (0.88)	16.6 (2.06)	<.001
Automated readability index, mean (SD)	10.4 (1.32)	8.91 (0.95)	16.1 (2.10)	<.001
Coleman-Liau index, mean (SD)	12.3 (1.48)	10.9 (0.86)	14.2 (1.58)	<.001
Letters per word, mean (SD)	5.14 (0.28)	4.90 (0.17)	5.35 (0.28)	.002
Syllables per word, mean (SD)	2.20 (0.11)	2.08 (0.08)	2.32 (0.11)	<.001
Words per sentence, mean (SD)	15.5 (3.18)	14.6 (1.64)	26.0 (3.29)	<.001
Complex words (%), mean (SD)	18.6 (3.94)	15.6 (2.67)	20.6 (4.40)	.02

## Discussion

This study provides exploratory evidence that Maria Ciência is capable of producing audience-adapted science communication materials that are perceived as clear, accessible, and linguistically appropriate across a range of stakeholders. Materials tailored for children and the general public were especially well received, while outputs for health managers showed greater variability, reflecting the distinct informational demands of this audience. The more critical feedback from original paper authors highlights the inherent challenge of balancing scientific precision with public accessibility.

Qualitative feedback from anonymous evaluators reinforced these trends, highlighting the clarity and perceived usefulness of the materials and offering constructive suggestions to further adapt tone and terminology for specific audiences. These results are encouraging, particularly in light of the urgent need to address health misinformation, which continues to erode public trust in science, hinder the implementation of health policies, and contribute to adverse health outcomes [1,2]. The COVID-19 pandemic brought the urgency of this issue into sharp focus, with widespread infodemics interfering with disease prevention efforts and amplifying avoidable harm [2], emphasizing the importance of tools that support effective science communication.

By enabling the production of trusted, audience-specific materials, Maria Ciência can complement existing strategies for combating misinformation, which traditionally rely on reactive fact-checking or broad public health campaigns [16]. Within the scope of this evaluation, Maria Ciência demonstrated superior conversational stability and contextual accuracy, particularly for sensitive audiences such as children, critical for fostering health literacy from an early age. Furthermore, previous studies have shown that GPT-based assistants specifically trained or configured with domain-relevant and ethically curated materials achieve higher performance and contribute to greater user trust and acceptance of the platform in public health and educational contexts [5,6]. The approach adopted by Maria Ciência, combining prompt engineering with thematic supervision, is consistent with these findings and reinforces the value of domain-specific configuration for science communication tools.

Beyond model performance, Maria Ciência was designed to reduce practical barriers that limit researchers’ engagement with public audiences. Despite growing recognition of science communication as a core responsibility, academic structures still prioritize publication in peer-reviewed journals over community outreach [3]. Researchers often lack time, institutional support, or training to translate their findings into accessible formats. The data presented here suggest that even minimal support from tools such as Maria Ciência can enable more scientists to participate meaningfully in public engagement by providing formats aligned with the needs of different audiences and reducing technical barriers to communication.

Complementing subjective evaluations, the readability analyses provide an objective lens through which to interpret these findings. Texts generated by Maria Ciência exhibited a deliberate balance between linguistic complexity and overall legibility. In some audience categories, modest increases in grade level, sentence length, or syllabic density were observed; however, these changes did not consistently translate into lower legibility classifications. Instead, they reflect structural adjustments aimed at preserving informational completeness rather than a loss of accessibility. Readability indices such as the Flesch reading ease, Flesch-Kincaid grade level, Gunning fog index, ARI, Coleman-Liau index, and Gulpease index are widely used in health communication research as proxies for accessibility and audience appropriateness [17,18]. Their combined use is particularly valuable for comparative analyses, as it allows systematic assessment of how different communication strategies modulate linguistic complexity while maintaining interpretability. Importantly, readability metrics are not end points: they function as proxies that can be linked to downstream outcomes such as comprehension, perceived credibility, and cognitive load, especially in settings where audiences must navigate competing narratives and misinformation. Lower linguistic complexity and shorter syntactic structures have been associated with improved comprehension and reduced cognitive load, particularly among audiences with limited health literacy, and may support trust-building when messages must compete with misleading narratives in digital environments [17,19,20]. Although readability alone cannot guarantee understanding or prevent misinformation, it provides an objective benchmark for whether outputs are likely to be approachable for nonspecialist readers and therefore serves as a useful complement to subjective quality ratings.

Direct comparisons with human-written science communication materials further contextualize these results. Human-authored texts consistently demonstrated substantially higher linguistic complexity across nearly all indices, including grade level, sentence length, and lexical density, reinforcing longstanding evidence that expert-driven communication frequently exceeds recommended readability thresholds for lay audiences. In contrast, both Maria Ciência and the base GPT-4.5 model produced texts that were systematically easier to read and more linguistically accessible. While both systems diverged from human writing in the same direction, Maria Ciência’s outputs were consistently simpler than those generated by the base model across most metrics. Together, these findings suggest that Maria Ciência does not indiscriminately simplify content but rather modulates textual structure in an audience-dependent manner, supporting improved accessibility without compromising overall legibility.

Differences observed for health managers are also conceptually expected. Communication for managerial or policy-facing audiences typically prioritizes actionability, decision relevance, and structured synthesis rather than simplified language alone. As a result, evaluation criteria that work well for general audiences may undercapture what makes output useful for managers or policymakers, and future evaluations should incorporate audience-specific criteria tailored to decision-making contexts.

From an implementation perspective, these characteristics position Maria Ciência as a practical support tool for knowledge translation workflows. Potential applications include integration into journal dissemination processes, public health communication teams, and institutional outreach initiatives, where rapid generation of audience-specific summaries can support timely and responsible dissemination of peer-reviewed evidence. Importantly, such integration should remain coupled with human oversight to ensure scientific fidelity and contextual appropriateness. For example, a journal or research group could (1) identify a newly accepted open-access paper, (2) generate a public-facing summary and a social media post using Maria Ciência, (3) generate a brief manager-facing version highlighting actionable implications, (4) conduct a rapid author or communications-team review for scientific fidelity and tone, and (5) disseminate the finalized outputs through institutional websites, press offices, and social channels. Of note, although not evaluated in this paper, Maria Ciência is also capable of generating images that can help in the dissemination of knowledge. This hypothetical pipeline illustrates how AI-assisted generation can reduce production time while preserving accountability through human review.

Recent advances in AI also point to future directions for audience-centered health communication tools that extend beyond the scope of this study. Federated learning frameworks, for example, have been increasingly explored in health care to enable collaborative model improvement across institutions while preserving data privacy and regulatory compliance [4]. In the context of health communication, such approaches may become relevant for incorporating region- or institution-specific linguistic and cultural patterns without requiring direct sharing of sensitive data. Similarly, developments in self-supervised learning highlight the potential of leveraging large volumes of unlabeled data to improve contextual understanding and adaptability of language models. In health care and related domains, self-supervised approaches have been proposed to enhance prediction and pattern recognition while reducing dependence on costly manual annotation [21]. Although not applied in the present work, these principles suggest possible future pathways for improving the adaptability and robustness of AI-assisted communication systems, particularly in settings where labeled training data are scarce or ethically constrained. Together, these developments emphasize that future research should explore how privacy-preserving and data-efficient learning paradigms can complement human oversight in responsible AI-driven health communication.

As AI-driven science communication tools gain increasing relevance worldwide, it is important to consider their potential for application across diverse international contexts. Although initially developed and evaluated in Brazilian Portuguese, the architecture and prompting framework of Maria Ciência, being built on the GPT platform, allow the tool to operate in over 50 languages with the same ethical and technical standards [14]. This provides immediate potential for multilingual deployment in global health communication efforts. However, while the tool can linguistically adapt to multiple languages, effective application across different regions also requires attention to cultural nuances, health literacy variations, and sociolinguistic differences. Linguistic translation alone is insufficient; local framing, terminology, and narrative styles must be carefully adapted to ensure meaningful engagement and to support health literacy and misinformation prevention across diverse public health ecosystems. Accordingly, future work should test culturally grounded adaptation workflows, ideally combining local expert review with community feedback.

Despite these strengths, this study has limitations. The number of evaluators per target audience was not standardized, and some subgroups, such as health managers and communication professionals, were underrepresented. Author evaluations, while informative, were based on subjective perceptions and not validated against formal scientific fidelity criteria. The evaluation also focused on perception and usability rather than long-term impacts on knowledge retention or behavioral outcomes. Third, children were not included as evaluators. As a result, evaluations of children-focused materials reflect adult perceptions and should be interpreted as preliminary. Furthermore, the tool was primarily tested in Brazilian Portuguese; while the underlying architecture is multilingual, additional research is needed to validate its performance in other languages and cultural contexts.

Even with these limitations, the evaluation provides a strong foundation to support the validity and utility of the tool. The systematic and transparent development process, combined with broad stakeholder engagement, underscores the potential of Maria Ciência to contribute meaningfully to health literacy and misinformation prevention. Its ability to outperform a baseline GPT model in contextual stability and accuracy further validates the importance of domain-specific configuration for public health applications. Future evaluations will adopt a stratified design in which materials are assessed exclusively by their intended target audiences, using validated instruments and balanced stakeholder representation.

In summary, Maria Ciência offers a promising avenue for enhancing knowledge translation and addressing the communication challenges posed by health misinformation. As international organizations and national governments have emphasized, combating the impacts of infodemics requires more than reactive correction. It necessitates proactive investment in tools and strategies that produce accessible, trusted, and culturally relevant information. By supporting the generation of audience-adapted materials and facilitating researcher engagement in public dialogue, Maria Ciência contributes to this broader effort to advance scientific literacy and strengthen public health resilience.

## Supplementary material

10.2196/78843 Multimedia Appendix 1 List of comments, and comparative accuracy and context stability evaluation table.

## Abbreviations

AI: artificial intelligence
ALT: analysis of language and text
ARI: automated readability index
