# Supplementary material for: Audience-Specific Health Communication: Mixed Methods Evaluation of the Maria Ciência AI-Assisted Knowledge Translation Tool
Source: JMIR Infodemiology. 2026 Mar 3;6:e78843. doi: 10.2196/78843 (PMC12978924; doi:10.2196/78843)
Supplement: Multimedia Appendix 1 — List of comments, and comparative accuracy and context stability evaluation table. [file infodemiology-v6-e78843-s001.docx]

**Supplementary Material**

**LIST OF COMMENTS**

**For Children:**

**Comment 1:**
This is a material with a simple, engaging, and creative approach.

**Comment 2:**
The text is easy to understand, but oversimplifies biological processes due to the adaptation for the target audience.

**Comment 3:**
Although the summary is well adapted for children using storytelling and narratives, I believe some parts could be simplified further. For example, the section addressing social inequalities might be too advanced for a younger audience, making the message harder to grasp. A reformulation using more accessible terms could help convey the idea without complicating the narrative. Also, I feel the text includes excess information that may not be useful for children and could be more direct.

**Comment 4:**
Very creative! I even use the platform to explain things to my patients.

**Comment 5:**
I think there could be more dialogue between Dr. Science and Leo instead of long narrator texts, but I found it very playful.

**Comment 6:**
It could be a little longer. Kids usually enjoy stories with more involvement and development.

**Comment 7:**
I found it very interesting, clearly it summarizes the article a lot but presents it in a way that's truly accessible for children.

**Comment 8:**
I thought the storytelling format aligns very well with the audience’s expectations.

**Comment 9:**
Well constructed; the adaptation for children aged 7 to 9 that I requested was very well executed by Maria Ciência. They managed to take a complex topic and simplify it in a magical and engaging way with detailed biology, without it being boring for kids.

**Comment 10:**
Well designed and simplified for children. The text is well adapted.

**Comment 11:**
I found it very understandable, both in terms of language and clarity of information.

**Comment 12:**
In the second paragraph, where it says who CAN abandon treatment, it may give the impression of permission for children, making them think it’s okay to stop treatment. It would be better to say that some patients tend to abandon treatment, and that the new tool aims to identify such profiles early and provide closer follow-up to ensure full treatment completion.

**Comment 13:**
Easy to understand.

**Comment 14:**
Very appropriate for the audience and very good!

**For health managers:**

**Comment 15:**
The text contains incorrect information. SITETB was not used for the analysis, and the study did not test other medications or their association with patient outcomes.

**Comment 15:**
Although it provides relevant insights, the text fails to address geographic disparities in coverage, an essential aspect for health managers. Understanding regional differences could guide improvements in test availability. This may have been omitted because these findings are only mentioned at the end of the results section (end of the PDF).
Additionally, the original article contains a table with five implications for health management that was also left out, possibly for the same reason.

**Comment 16:**
The text is well written and targeted at health managers. However, it misses some important points. While it correctly highlights consumption habits and vulnerable populations, it fails to mention another key finding: patients with TB relapse or retreatment also have a high risk of being lost to follow-up or treatment failure.
Moreover, some details like the study name, journal page link, and DOI are incorrect in the GPT-generated text.

**Comment 17:**
Disconnected words, results not well explained, but it gives a good direction for public policy.

**Comment 18:**
The text for managers is quite understandable. It highlights the key points of the article and helps generate new strategy ideas. The recommendations proposed by Maria Ciência are feasible and relevant to management. I really liked it. Congrats!

**Comment 19:**
It doesn’t seem targeted toward health managers.

**Comment 20:**
There is an error in the description of RePORT: "RePORT-Brazil is a multicenter cohort of people with pulmonary TB, started in 2015, with participants from four urban centers (Manaus, Recife, Rio de Janeiro, and Salvador)."

**Comment 21:**
The text is well written and targeted at the intended audience, but it could include more information about the study design and its results. It would also be important to mention the study’s limitations. For health managers, these are useful insights to decide which studies can inform public policy.

**Comment 22:**
I think the text is well written and appropriately aimed at the audience. However, I missed a section describing the article’s main limitations. This is important for health managers to determine which study findings are most suitable for supporting policies.

**Comment 23:**
I believe the text is well written and targeted at the audience, but I missed a section discussing the article’s limitations.

**Comment 24:**
I think the text is well written and appropriately targeted.

**For social media:**

**Comment 25:**
It provides incorrect information about evaluating other medications and their association with outcomes.

**Comment 26:**
I believe some issues are important to highlight: The LinkedIn post should adopt a more technical and professional tone compared to Instagram. It seems Maria Ciência struggled to adjust the language for each social media platform; As a result, important aspects were omitted, such as regional disparities in test adoption. For instance, the study showed peripheral regions, typically with less healthcare access, benefited the most (e.g., the North region had the highest coverage and impact), but this wasn't mentioned; Some sections introduced topics not in the original article, such as a focus on vulnerable populations and the claim that the analysis involved 3,200 municipalities.

**Comment 27:**
The LinkedIn text is excellent. However, the Instagram summary could be improved with a bit more detail in the caption, perhaps including a brief summary of the study to help readers understand the main findings.

**Comment 28:**
It addresses a very important, often stigmatized topic in a clear and objective way, fulfilling its intended purpose.

**Comment 29:**
Although well written and adapted to the audience, Maria Ciência tends to oversummarize information, even though it’s possible to present content in an accessible way with a bit more detail. This limitation is understandable, as the content should undergo human review before social media dissemination. Also, the image generated had a top section cut off, not invalidating it, but potentially challenging for social media editing.

**Comment 30:**
The chatbot provided many good insights, but it was a bit too brief. That’s likely to make it easier to understand for the general public.

**Comment 31:**
I just think it could’ve emphasized the importance and relevance of the test more, especially on Instagram, where most readers are laypeople.

**Comment 32:**
I think it could have provided more developed suggestions for Instagram carousel images.

**Comment 33:**
Very well written and clearly explained. Great for reader understanding.

**Comment 34:**
When saying: “People who had COVID and then got TB,” some might interpret TB as a COVID sequel. It’s better to clarify that people who had COVID and later contracted TB should be more vigilant.

**Comment 35:**
I think the LinkedIn post is great, but the Instagram post would benefit from a more detailed caption. Currently, it lacks study information.

**Comment 36:**
The Instagram post only describes the results for 3 out of the 4 age groups analyzed and doesn’t mention the TB in Adults section. The LinkedIn text is complete.

**Comment 37:**
Perfect.

**Comment 38:**
Very good and richly informative democratic in tone.

**For general population:**

**Comment 39:**
The text suggests iron supplementation, which an AI should not recommend. It may be harmful, especially in the TB context.

**Comment 40:**
The text suggests the use of medication, which should not happen.

**Comment 41:**
The text is very well written, especially for a lay audience. However, it contains two small methodological errors. First, the article never says “more than 3,200 Brazilian municipalities” were analyzed. Second, the study period ranged from 2011 to 2022 (the text says 2010–2020).

**Comment 42:**
The material is clear, written objectively, and structurally organized.

**Comment 43:**
In a few parts, there is information overload for the general public. That’s why I gave the “Language Appropriateness” four stars.

**Comment 44:**
The article summary is clear and concise. Easy to understand for non-health readers. Using terms like “pre-diabetes,” “blood sugar,” and “long treatment” made the topic more accessible.

**Comment 45:**
Very good, very educational! The use of bullet points makes the reading more dynamic and simple.

**Comment 46:**
Clear and objective!

**Comment 47:**
Excellent.

**Comment 48:**
The chosen article shows the main advances and stages of biomarker research and the existing barriers to validation and implementation, while proposing solutions to accelerate effective TB detection.

**Comment 49:**
Excellent.

**Comment 50:**
The material clearly and directly relates the diseases and shows how diabetes complicates TB, requiring close attention to improve outcomes and treatment.

**Comment 52:**
Super clear and easy to follow. Highly relevant topic.

**Comment 53:**
Material with clear and effective language.

**Comment 54:**
Clear, providing high-quality information.

**Comment 55:**
The text is concise and the topics are well explained.

**Comment 56:**
The text is brief and the topics are well explained.

**Comment 57:**
The article is more targeted toward health professionals. The most impactful message for the general public is the existence of a rapid test. This should be the focus.

**Comment 58:**
One of the best texts I’ve read. Communicates and explains very well.

**Comment 59:**
Text is similar to ART 04, but the study’s scope is broader, I didn’t find it as strong as the previous one.

**Comment 60:**
I found the text weaker than the previous one, maybe due to the smaller research scope.

**Comment 61:**
Due to low relevance for the general public, the text doesn’t communicate well with its audience. It should focus more on the drug’s use and benefits than the discovery itself.

**Comment 62:**
I think the text is too informative and doesn’t connect well with the target audience.

**Comment 63:**
Great text! Short and communicates well with the general public, with high relevance for the broader population.

**Comment 64:**
Similar to ART 09, but unlike it, this one doesn’t engage well with the target audience.

**Comment 65:**
Very enlightening.

**Comment 66:**
Very well written and informative combined with an eye-catching image, it will reach a wide audience.

**Comment 67:**
Perfect!

**Comment 68:**
The material is very interesting, congrats. However, some terms are too technical (e.g., rapid test, bacterial load, cycle threshold). It might be worth simplifying the language for non-experts.

**Table S1.** Average (SD) evaluation scores of Comparative accuracy and context stability evaluation.

| **Target Audience** | **Evaluator** | **Establishment of a context** | **Continuity of conversation** | **Interruption of context** | **Return to context** |  |
| --- | --- | --- | --- | --- | --- | --- |
|  |  |  |  |  |  |  |
| **For general public, mean (SD):** | *Maria Ciência* | 4.75 (0.50) | 5.00 (0.00) | 4.75 (0.50) | 4.75 (0.50) |  |
|  | GPT base | 4.25 (0.96) | 4.25 (0.50) | 4.25 (0.50) | 4.75 (0.50) |  |
| **For children, mean (SD):** | *Maria Ciência* | 4.66 (0.58) | 4.66 (0.58) | 5.00 (0.00) | 4.66 (0.58) |  |
|  | GPT base | 3.25 (0.50) | 3.00 (0.82) | 3.75 (0.50) | 4.50 (0.60) |  |
| **For health managers, mean (SD):** | *Maria Ciência* | 4.75 (0.50) | 5.00 (0.00) | 4.50 (0.58) | 4.50 (0.58) |  |
|  | GPT base | 4.50 (0.58) | 4.50 (1.00) | 4.25 (0.50) | 5.00 (0.00) |  |
| **For social media, mean (SD):** | *Maria Ciência* | 4.50 (0.58) | 4.75 (0.50) | 5.00 (0.00) | 4.00 (0.00) |  |
|  | GPT base | 4.50 (0.58) | 4.50 (0.58) | 4.50 (0.58) | 5.00 (0.00) |  |

**Table note**: This table presents the central tendency and dispersion (mean [standard deviation, SD]) of four accuracy metrics: establishment of a context (A), continuity of a conversation (B), interruption of context (C) and return to context (D), across four target‐audience contexts (“For children,” “For health managers,” “For social media,” “For general public”). Within each context, scores are shown separately for each model (“Maria Ciência” and “GPT base”). All ratings were provided on a five‐point scale ranging from 1-5.
